# An In Vivo and In Vitro Evaluation of the Mutual Interactions between the Lung and the Large Intestine

**DOI:** 10.1155/2013/695641

**Published:** 2013-07-21

**Authors:** Lei-Miao Yin, Guang-Quan Zhang, Xing-Ke Yan, Yu Wang, Yu-Dong Xu, Yong-Qing Yang

**Affiliations:** Shanghai Research Institute of Acupuncture and Meridian, Shanghai University of Traditional Chinese Medicine, Shanghai 200030, China

## Abstract

One of the most important theories of the traditional Chinese medicine is the exterior-interior relationship between the lung and the large intestine; so far, little direct experimental evidence has been reported to support such relationship. Here we for the first time investigated the mutual interactions between the lung and the large intestine by examining the relevancies between the pulmonary functions and the rectal resting pressure in the rat models of asthma and constipation. We also evaluated the effects of the lung homogenate and the large intestine homogenate on the isolated large intestine muscle strip and the isolated tracheal spiral, respectively. Our results showed that the pulmonary resistance and pulmonary compliance were closely related to the rectal resting pressure in the asthmatic rat model, while the rectal resting pressure was much correlated with the pulmonary resistance in the rat model of constipation. Moreover, it was shown that the lung homogenate could specifically contract the isolated large intestine muscle strip. Overall, this study provided new lines of evidence for the theory and highlighted the potential application in the treatment of the corresponding diseases.

## 1. Introduction

The theory of the “exterior-interior relationship between the lung and the large intestine” originates from the Yellow Emperor's Classic of Internal Medicine (Huang Di Nei Jing), which is one of the most important governing principles of traditional Chinese medicine in the clinical application [1]. According to the theory, the lung meridian communicates with the large intestine meridian, which creates an exterior-interior relationship and influences each other specifically [2]. The lung diseases affect the condition of the large intestine except for the symptoms of nose and throat, while the disturbances of the large intestine could cause the pulmonary and bronchial dysfunctions. The lung is the essential respiration organ while the large intestine absorbs water and passes the wastes out of the body; obviously the two organs are anatomically separated; however, there are several aspects of evidence for the special relationship between the lung and the large intestine in both physiology and pathology.

The possibility of the mutual interaction between the lung and the large intestine was demonstrated by the same origin, the interactive pathophysiologic relationship, and the common effectors molecules. From the developmental point of view, the foregut was the common developmental structure of the lung and the large intestine, which suggested that there were similar regulations in the critical biological processes, such as the cellular apoptosis, mutations, communication, proliferation, and differentiation [3, 4]. Besides, the structures of bronchus and intestine were both characterized by the columnar epithelia with goblet cells and submucosal glands, which provided a basis for the development of inflammatory changes in both bronchus or bowel diseases under the same circumstance [5].

In the normal condition, the lung maintains the movement and gas emission of the large intestine by respiratory movement, while the large intestine assisted the lung to conduct proper breathing function [6]. In pathological conditions, the disorders of the lung and the large intestines can affect each other. The epidemiological survey showed that lung and large intestine cancers were closely associated with the industrialization and the related factors [7]. Large intestine carcinoma is usually accompanied by cough and shortness of breath because of endo-bronchial metastases [8]. Alveolar leakage and airway inflammation were found during intestinal ischemia by using laser confocal microscope [9]. Similarly, the intestinal permeability was increased in children with asthma [10]. It is reported that 28.5% of patients with inflammatory bowel diseases had abnormal pulmonary function tests, and 22% of patients had small airway obstruction or bronchiectasis via high-resolution CT screening [11]. The gut-derived factors contributed to burn-induced lung injury and were related to subsequent respiratory failure [12]. In multiple organ dysfunction syndrome (MODS), the digestive tract symptoms often appeared after acute lung injury [13]. The activation of neutrophils in blood released a large number of enzymes and cytokines and caused inflammatory reaction in both lung and large intestine [14], which suggested that active components in the blood circulation and lymphokinesis may contribute to the relationship between the two organs.

A serial of common effector molecules were found in various pathophysiologic conditions in both lung and intestine, such as pulmonary surfactants family, vasoactive intestinal peptide, pulmonary thromboxane A2, sIgA (soluble immunoglobulin A), and cholecystokinin. The pulmonary surfactants played a key role in the evolution of air breathing [15]; however, intestinal surfactants had an earlier origin which could perform a variety of functions after the secretion of enterocyte [16, 17]. It is reported that the pulmonary surfactant proteins A and D were expressed in both pulmonary and gastrointestinal epitheliums [17], which extended the concept of intestinal surfactant and underlined its close relationships with pulmonary surfactant [18, 19]. It is recognized that the onset of asthma and airway responsiveness were closely associated with decrease of vasoactive intestinal peptide (VIP), which influenced the endogenous oxidant/antioxidant balance and the relaxation of the intestine [20]. Through the release of pulmonary thromboxane A2 (TxA2), intestinal reperfusion induced pulmonary vaso-constriction and increased pulmonary microvascular permeability [21]. After intestinal ischemia and reperfusion, the release of nitric oxide (NO) from the pulmonary vascular endothelium and the airway smooth muscle contraction were impaired, which may contribute to the respiratory failure [22]. The trefoil factor family domain peptides (TFF), responsible for the protection and repair of the intestinal epithelium, were expressed much higher in respiratory tract than in colon tissue and found to be closely associated with lung function [23]. Pathogenic effector Th17 cells were considered to be the common pathogenetic basis in both intestinal and respiratory tracts [24].

The above-mentioned facts provided preliminary scientific evidence for the close relationship between the two organs; however, direct experimental proofs are still lacking. Our study aims to investigate the mutual interactions between the lung and the large intestine by examining the relevancies between the pulmonary functions and the rectal resting pressure in the rat models of asthma and constipation, evaluate the effects of the lung homogenate and the large intestine homogenate on the isolated large intestine muscle strip and the isolated tracheal spiral, respectively, which could provide not only solid experimental evidence for the special relationship between lung and large intestine but also clues for solving complex clinical problems.

## 2. Method

### 2.1. Animals

Male Sprague-Dawley (SD) rats (4 weeks old, 110–130 g, SLAC Laboratory Animal Co. Ltd., Shanghai, China) were raised in a pathogen-free rodent facility and provided with food and water *ad libitum*. Rats were kept in animal facilities approved by the Shanghai Committee for Accreditation of Laboratory Animal, and the animal experiment conformed to the regulations of the State Science and Technology Commission.

### 2.2. The Rat Model of Asthma

Rats were randomly divided into two groups (*n* = 8 each): control and asthmatic model groups. The protocol of SD rat model of asthma was described as previously [25]. Briefly, rats were sensitized with 1 mg ovalbumin (OVA) precipitated with 10 mg of aluminum hydroxide gel in 1 mL normal saline with intraperitoneal injection. The sensitized rats were challenged two weeks later with 1 mL/kg of 5% OVA in normal saline by injection into the external jugular vein over 10 s. Control rats were sensitized and challenged with normal saline instead of OVA.

### 2.3. The Rat Model of Constipation

Rats were randomly divided into two groups (*n* = 8 each): control and constipation model groups. The protocol of rat model of constipation was modified from Shan et al. [26]. Briefly, after fasting for 24 h with free access to water, rats were treated with compound diphenoxylate (10 mg/kg body weight, once a day, for four days) in 1 mL normal saline through intragastric administration, and the time of first defecation with the charcoal meal was recorded. Number and weight of feces were recorded 12 h after the administration. Control rats were treated with normal saline instead of compound diphenoxylate.

### 2.4. Measurements of the Pulmonary Functions and the Rectal Resting Pressure

When measuring the pulmonary function, a rat under anesthesia was placed on a wood plate in the supine position. A heater was controlled pneumotachograph which connected to a differential pressure transducer (600D-011, AutoTran, USA) was gently inserted into the trachea, and the tidal flow was determined. To measure transpulmonary pressure, a water-filled tube which coupled to a pressure transducer (PT14MX, Jialong Teaching Equipment, Shanghai) was inserted into the esophagus to the level of the midthorax. The pneumotachograph tidal flow signal was integrated with time to obtain tidal volume, which was continuously recorded for 30 min. The measurement of rectal resting pressure was modified from Hancock [27]. The tube (diameter = 2.5 mm) with a balloon at the front end was inserted slowly into anus for 3.5 cm, which connected with pressure transducer (PT14MX, Jialong Teaching Equipment, Shanghai). Twenty min after the tube insertion, rectal resting pressure was recorded by the SMUP-B biological signal analysis system for 30 min.

### 2.5. The Preparation of the Tissue Homogenates

After rats were sacrificed, the lung (including trachea), heart, small intestine (total ileum), and large intestine (total colon) were taken out quickly and respectively. After washing with normal saline and removal of fat, the tissues were weighed and mixed with normal saline at the ratio of 1 : 4. By using electric glass homogenizer machine in ice bath, the tissues were turned into homogenates. After centrifugation at 3500 r/min for 20 min, the supernatants were kept. The protein concentrations of the homogenates of the lung, heart, large intestine and small intestine, were all adjusted to 5 mg/mL, BSA (5 mg/mL) and normal saline were served as control.

### 2.6. The Preparation of the Tracheal Spiral and the Large Intestine Muscle Strip

The trachea and large intestine were immediately removed after the sacrifice of rats. The adherent connective tissue and fat on the surface were all removed, and the tissues were put into a Petri dish full of Krebs'-Henseleit (KH) solution (NaCl 118.0 mmol/L; KCl 4.7 mmol/L; CaCl_2_ 2.5 mmol/L; MgSO_4_ 1.2 mmol/L; NaHCO_3_ 25.0 mmol/L; KH_2_PO_4_ 1.2 mmol/L; glucose 10.0 mmol/L). A tracheal segment (approximately 20 mm in length from thyroid cartilage to bifurcation of tracheas) was isolated and then suspended in a 80 mL organ bath by two stainless-steel wires (0.3 mm diameter). The 2.5 cm long large intestine was quickly cut (20 mm away from the lower end of the cecum). One end was fixed to the bottom of the organ bath, whereas the other was connected to the pressure transducer (PT14MX, Jialong Teaching Equipment, Shanghai) for the measurement of isometric tension. The tracheal spiral and the large intestine muscle strip were set up vertically under a tension of 1.5 g, which were maintained in the KH solution. The organ bath was kept at 37.0 ± 0.5°C and continuously gassed with 95% O_2_ and 5% CO_2_. The measurement of the isometric tension was recorded, and the tension changed in 10 min was analyzed. Each tracheal spiral and large intestine muscle strip were used only one time.

### 2.7. Data Analysis

All data are expressed as the mean ± SD. The Student's *t*-test was used to analyze the significance of the pulmonary resistance, the pulmonary compliance, and the rectal resting pressure between the two groups in vivo. The relationship between two variables was identified by the linear regression analysis. One-way ANOVA (analysis of variance) followed by the least significant difference (LSD) test for post hoc analysis was used to analyze the significance of tension among the different groups in vitro. The *P* value that was lower than 0.05 was considered significant.

## 3. Results

### 3.1. The Pulmonary Functions Were Closely Related to Rectal Resting Pressure in the Rat Models of Asthma and Constipation

There was no significant relationship between rectal resting pressure and pulmonary resistance in the control rat within 30 min (*r* = −0.032,  *P* > 0.05), and there was also no significant relationship between rectal resting pressure and pulmonary compliance within 30 min (*r* = −0.050,  *P* > 0.05). In asthmatic rat, the pulmonary resistance was significantly increased at 3–7 min (*P* < 0.05, Table 1); the pulmonary compliance was significantly decreased at 3–10 min (*P* < 0.05), which suggested the successful establishment of asthma model. There was a significant difference in the rectal resting pressure at 2 min between the asthmatic model and the control group (*P* < 0.05). Three to seven min after OVA challenge, the rectal resting pressure was decreased and had a negative relationship with pulmonary resistance within 30 min (*r* = −0.423,  *P* < 0.05). Meanwhile, the rectal resting pressure has a positive relationship with pulmonary compliance within 30 min (*r* = 0.711,  *P* < 0.05), which demonstrated that the changes of the pulmonary resistance and pulmonary compliance were closely associated with the level of the rectal resting pressure in the asthmatic model.

Twelve h after compound diphenoxylate administration, the number of stool of rats was significantly decreased by 77.23% when compared to that of the control group (*P* < 0.05). The weight of stool was significantly decreased in rats in the group of compound diphenoxylate administration by 76.01% (*P* < 0.05), which suggested the successful establishment of the constipation model. There was no significant difference in the rectal resting pressure between the constipation model and the control group in 10 min (*P* > 0.05), although the rectal resting pressure of the constipation rats had a tendency to decrease. The pulmonary resistance had a tendency to increase (*P* > 0.05) but had no significant relationship with the rectal resting pressure within 30 min (*r* = 0.063,  *P* > 0.05). However, the pulmonary compliance of the constipation model was significantly decreased at 1-2, 4–7, and 9-10 min (*P* < 0.05, Table 2) and had a significant positive relationship with the rectal resting pressure within 30 min (*r* = 0.663, *P* < 0.05), which suggested that the constipation and the breath difficulty happened at the same time, and the change of the pulmonary compliance was closely associated with the level of the rectal resting pressure in the constipation model.

### 3.2. Measurements of the Isometric Tensions

The different levels of the acetyl choline (Ach) were used to test the contractive effect on the isolated large intestine muscle strip and the isolated tracheal spiral, and the 70%–75% of the maximal contractile response was chosen and examined in order to avoid the maximum of the concentration response and allow for adequate extension [28]. In the study, the addition of 0.02 mg/L and 20 mg/L Ach produced 70%–75% of the maximum contraction of the isolated large intestine muscle strip and the isolated tracheal spiral, respectively, which suggested the successful establishment of the in vitro testing system and demonstrated that the sensitivity of isolated large intestine muscle strip was about 1000 times higher than that of the isolated tracheal spiral.

**Figure 1:**
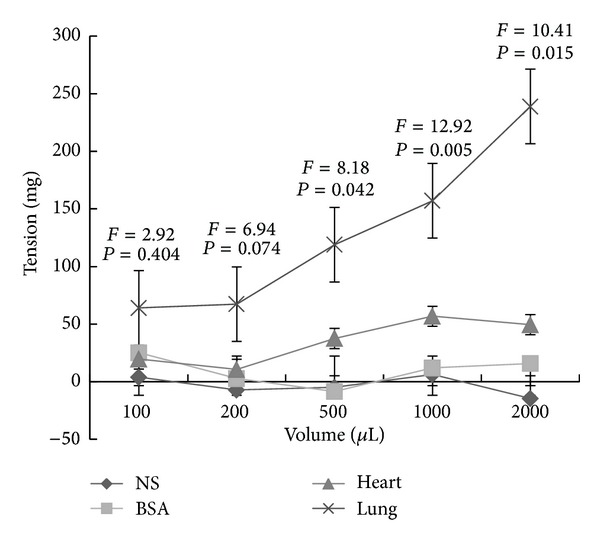
The effects of the lung homogenate, the heart homogenate, BSA, and normal saline on the isolated large intestine muscle strip. The addition of 100, 200, 500, 1000, and 2000 *μ*L of the 5 mg/mL lung homogenate, heart homogenate, BSA, and normal saline into the in vitro testing system of the isolated large intestine muscle strip. The addition of the lung homogenate could significantly contract the isolated large intestine muscle strip at the volume of 500, 1000, and 2000 *μ*L (*P* < 0.05).

Different volumes of the tissue homogenates were added separately into the bath of the large intestine muscle strip, and, with the increase of the volume, the contraction response was recorded. The addition of the 100, 200, 500, 1000, and 2000 *μ*L of the 5 mg/mL heart homogenate, BSA, and normal saline had no significant effects on the isolated large intestine muscle strip (*P* > 0.05, Figure 1); however, the addition of 500, 1000, and 2000 *μ*L of the 5 mg/mL lung homogenate could significantly contract the isolated large intestine muscle strip (*P* < 0.05), which demonstrated the special effect of lung homogenate on the isolated large intestine muscle strip. In the group of 500 and 1000 *μ*L addition, the tension induced by the lung homogenate was 3.1 and 2.8 times greater than that of the heart homogenate (*P* < 0.05). In the group of 2000 *μ*L addition, the tension induced by the lung homogenate was the maximal and 4.8 time greater than that of the heart homogenate (*P* < 0.05).

**Figure 2:**
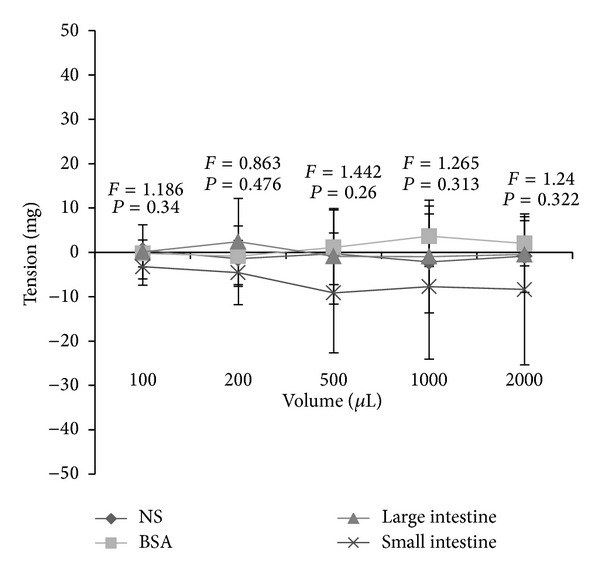
The effects of the large intestine homogenate, the small intestine homogenate, BSA, and normal saline on the isolated tracheal spiral. The addition of the 100, 200, 500, 1000, and 2000 *μ*L of the 5 mg/mL large intestine homogenate, small intestine homogenate, BSA, and normal saline had no significant effects (*P* > 0.05) on the isolated tracheal spiral.

The addition of the 100, 200, 500, 1000, and 2000 *μ*L of the 5 mg/mL large intestine homogenate, small intestine homogenate, BSA, and normal saline had no significant effects (*P* > 0.05, Figure 2) on the isolated tracheal spiral, which may be due to the low sensitivity of the isolated tracheal spiral.

## 4. Discussion

The data showed that the pulmonary resistance and pulmonary compliance were closely related to the rectal resting pressure in asthmatic rat model, and the rectal resting pressure was closely associated with pulmonary resistance in the rat model of constipation. The lung homogenate could specifically contract the isolated large intestine muscle strip in vitro. These results suggested that the lung and large intestine had a specific relationship between each other, which may provide clues for a further study.

The common active proteins and the corresponding receptors, such as sIgA and cholecystokinin, may account for the underlying mechanism of lung and large intestine mutual interaction. sIgA exists specifically in both lung and intestinal tissues and is considered to be the common mucosal immune molecules base [29]. The polymeric immunoglobulin receptors for IgA were also found expressed in lung, bronchi, gut, and so on, which was involved not only in the antigen-antibody complex recognition but also in various signal transduction under different conditions [30]. Another important neuropeptide cholecystokinin (CCK), which is expressed in the gastrointestinal nervous system, was now found widely distributed in trachea and alveoli [31]. CCK played a role in protecting gastric mucosa and stimulating the digestion of nutrition, and now totally two types of CCK receptors have been identified [32]. Recent studies have reported that the sIgA and CCK and their corresponding receptors may be closely associated with the specific relationship between the lung and large intestine. It is reported that the CCK2 receptor with retention of intron 4 (CCK2Ri4sv) was now a marker of specific gastrointestinal and lung tumors [33]. In CCR3 knockout mice model, the eosinophil recruitment in the lung was decreased while the number of intraepithelial mast cells was increased in the trachea after OVA sensitization and allergen challenge [34]. Study demonstrated that the expression of CCK receptors and cytokine productions on peripheral blood and bronchoalveolar lavage fluid (BALF) in asthma patients were significantly increased [35]. Modern research has confirmed that both the lung and large intestine were endocrine organs and could synthesize a serial of active substances when receiving signals from the internal and external environments. Ovalbumin (OVA) is widely used as a reference allergen to induce allergic asthma, which can cause the imbalance of CD4+ T lymphocytes, secretion of cytokines, and immune inflammation [36]. In our rat model of OVA-induced asthma, the expressions of immune-related proteins, like sIgA and CCK, could be changed in asthmatic lung; meanwhile the corresponding receptors were subsequently altered in the large intestine; in this way the pathological condition of lung may influence the status of large intestine and vice versa.

The intestine muscle strip is frequently used as an evaluation tool for the intestinal smooth muscle function, and the behavior of the intestines varies among different species. It is showed that the 0.01 mg/L Ach evoked the contraction of human colon [37], which was two times more sensitive than that of the rat intestine. The level of 0.03 mg/L Ach caused the contraction of the longitudinal muscle strip which was obtained from guinea pig ileum [38]. It is reported that 0.1–10 mg/L Ach contracted the longitudinal and circular muscle strips of rabbit while 0.1 mg/L atropine blocked the effect of 10 mg/L Ach [39]. In our large intestine muscle strip of rat, the level of 0.02 mg/L Ach caused about 70%–75% of the maximum contraction, and this data was consistent with the previous studies, which provided a suitable testing system for the in vitro research.

A serial of pharmacological agents have been tested in the model of the intestine muscle strip. It is reported that the 10–50 *μ*m ATP transmural stimulation caused relaxations of the rat duodenum and ileum [40]. Exogenous addition of 20 ng/mL IL-6 increased the contraction of the circular muscle strip of colon and suggested that the contraction induced by IL-6 may be due to the acting on the gut's nervous system [41]. As an antagonist of the muscarinic receptors, MB327 showed a fully reversible smooth muscle relaxing effect at lower concentrations in a rat jejunum smooth muscle model [42]. In our in vitro study, the addition of the lung homogenate contracted specially the isolated large intestine muscle strip, which suggested that the lung homogenate might contain active proteins that could bind to the corresponding receptors in the large intestine and carried out biological functions. Besides, it is also known that the interstitial cells, such as smooth muscle cells and the interstitial cell of Cajal (ICC), could release Ach by transmural stimuli and the activation of the cholinergic fibers [43, 44]. In our study, the addition of tissue homogenates may induce a small amount of Ach release of the large intestine and lead to the contraction. The study provided a foundation for further research, and, if a specific protein and receptor could be well identified in the future, it may contribute to the medical science and well reflect the biological value of traditional Chinese medicine.

## 5. Conclusion

In the current study, we showed that the changes of the pulmonary functions were closely related to the rectal resting pressure in the rat models of asthma and constipation, and the lung homogenate could significantly contract the large intestine muscle strip. It provided new lines of evidence for the “exterior-interior relationship between the lung and the large intestine” and highlighted the use of this theory in the treatment of the corresponding diseases in the future.

## Figures and Tables

**Table 1:** The pulmonary compliance, the pulmonary resistance, and the rectal resting pressure of the control and asthmatic model of rats.

Items	Groups	Min 1	Min 2	Min 3	Min 4	Min 5	Min 6	Min 7	Min 8	Min 9	Min 10
Pulmonary compliance (mL/kPa)	Control	−0.030 ± 0.001	−0.026 ± 0.001	−0.006 ± 0.001	−0.040 ± 0.001	−0.047 ± 0.001	0.020 ± 0.001	0.030 ± 0.002	−0.021 ± 0.001	−0.100 ± 0.002	−0.040 ± 0.002
	Asthma	−0.300 ± 0.007	−0.620 ± 0.010	−0.867 ± 0.010*	−0.806 ± 0.009*	−0.862 ± 0.010*	−0.866 ± 0.009*	−0.809 ± 0.009*	−0.713 ± 0.007*	−0.809 ± 0.010*	−0.788 ± 0.010*

Pulmonary resistance (kPa/mL/s)	Control	−0.001 ± 0.001	−0.001 ± 0.002	−0.001 ± 0.002	−0.001 ± 0.002	−0.002 ± 0.002	−0.001 ± 0.005	−0.001 ± 0.004	−0.001 ± 0.004	0.001 ± 0.008	0.001 ± 0.007
	Asthma	0.012 ± 0.012	0.029 ± 0.029	0.112 ± 0.033^#^	0.186 ± 0.028^#^	0.109 ± 0.029^#^	0.082 ± 0.030^#^	0.060 ± 0.017^#^	0.018 ± 0.015	0.014 ± 0.010	0.008 ± 0.006

Rectal resting pressure (kPa)	Control	−0.050 ± 0.142	−0.114 ± 0.107	−0.075 ± 0.149	−0.049 ± 0.070	−0.018 ± 0.147	−0.076 ± 0.115	−0.028 ± 0.113	−0.009 ± 0.093	−0.026 ± 0.177	0.022 ± 0.108
	Asthma	0.197 ± 0.401	0.013 ± 0.108^&^	−0.032 ± 0.099	−0.062 ± 0.107	−0.058 ± 0.130	−0.047 ± 0.107	−0.075 ± 0.157	−0.008 ± 0.116	−0.046 ± 0.196	−0.096 ± 0.169

Data were shown as mean ± SD (*n* = 8). The values of pulmonary resistance in the table were expressed as differential values subtracted from the corresponding baseline values. Statistical comparisons were made by the Student's *t*-test. *P < 0.05, compared to control; ^#^ *P* < 0.05, compared to control; ^&^ *P* < 0.05, compared to control.

**Table 2:** The pulmonary compliance, the pulmonary resistance, and the rectal resting pressure of control and constipation model of rats.

Items	Groups	Min 1	Min 2	Min 3	Min 4	Min 5	Min 6	Min 7	Min 8	Min 9	Min 10
Pulmonary compliance (mL/kPa)	Control	0.083 ± 0.021	0.080 ± 0.017	0.080 ± 0.016	0.081 ± 0.017	0.077 ± 0.020	0.080 ± 0.016	0.080 ± 0.018	0.074 ± 0.022	0.077 ± 0.018	0.084 ± 0.020
	Constipation	0.059 ± 0.007*	0.057 ± 0.007*	0.065 ± 0.015	0.058 ± 0.006*	0.059 ± 0.005*	0.059 ± 0.007*	0.059 ± 0.006*	0.058 ± 0.005	0.059 ± 0.006*	0.058 ± 0.006*

Pulmonary resistance (kPa/mL/s)	Control	0.096 ± 0.018	0.095 ± 0.016	0.096 ± 0.016	0.097 ± 0.015	0.097 ± 0.015	0.112 ± 0.043	0.098 ± 0.017	0.095 ± 0.020	0.088 ± 0.010	0.086 ± 0.008
	Constipation	0.093 ± 0.012	0.093 ± 0.014	0.096 ± 0.017	0.098 ± 0.018	0.098 ± 0.019	0.094 ± 0.017	0.096 ± 0.019	0.095 ± 0.017	0.096 ± 0.017	0.096 ± 0.015

Rectal resting pressure (kPa)	Control	0.072 ± 0.099	0.067 ± 0.092	0.048 ± 0.027	0.051 ± 0.023	0.063 ± 0.068	0.048 ± 0.017	0.077 ± 0.082	0.082 ± 0.078	0.102 ± 0.127	0.068 ± 0.073
	Constipation	0.019 ± 0.020	0.052 ± 0.066	0.055 ± 0.072	0.034 ± 0.046	0.069 ± 0.071	0.053 ± 0.073	0.042 ± 0.070	0.089 ± 0.101	0.056 ± 0.069	0.029 ± 0.047

Data were shown as mean ± SD (*n* = 8). The values of pulmonary resistance in the table were expressed as differential values subtracted from the corresponding baseline values. Statistical comparisons were made by the Student's *t*-test. *P < 0.05, compared to control.
